# Supplementary material for: Patterns of Intron Gain and Loss in Fungi
Source: PLoS Biol. 2004 Nov 30;2(12):e422. doi: 10.1371/journal.pbio.0020422 (PMC532390; doi:10.1371/journal.pbio.0020422)
Supplement: Table S1 — Also available at http://genes.mit.edu/NielsenEtAl/. (4.3 MB ZIP). [file pbio.0020422.st001.zip › NielsenEtAl/html/1290.html]

AN1082.1.NCU03713.1.MG08146.1.FG05143.1


```
 CLUSTAL W (1.82) Multiple Sequence Alignments - Introns Inserted


Sequence 1: NCU03713.1	556 aa
Sequence 2: MG08146.1	552 aa
Sequence 3: FG05143.1	547 aa
Sequence 4: AN1082.1	511 aa
Alignment Length: 567 aa
Number Identitical Residues: 255 aa
Alignment Score (without introns) 13231


MG08146.1 	MRRTSVSLPTNNKVARDPYEKRARY---EANQREVGLFEKITEA-MSQARQTRWFKTLAV
NCU03713.1	MRRTSVSLPT-KHVAHDPHEKPDRYRRDQHNQEPASVFIRMQNAWMSQSQKARYLKTGAI
FG05143.1 	-MRTPVSPTSSKLPGFDPHEKPDRY---KFKPKRQGIIGRMKESWMTQSQRTRWIKTAAI
AN1082.1  	---------------------------------------------MTQSQRSRYIKTGAI
          	                                             *:*::::*::** *:

MG08146.1 	LVVILFLFFFLSPSGGVDLYKS-~GV1PSAASPNS~GQAPADASYGTDKCTRSFSKDKPI
NCU03713.1	VFAVLFLFYLFSPSG-VEISGAD~GG1PSQG--NQ~GQVPSDSSYGTDRCTRSFSKEKPI
FG05143.1 	VFAIVTLFYFISPKG-VEVYHEV1TK~PAHA-INN~GQSPSDSSYGTDRCAKSFSKDKPI
AN1082.1  	VSALLLILFWLSPSR--------~-G~TGYSGRPG1SQLSAGGSSPTTKCSKPYDSSKPL
          	: .:: ::: :**.             .. ..    .* .:..*  * :*::.:...**:

MG08146.1 	VQYVLMIDAGSTGSRIHVYKFNNCGAAPELENEV-FKQTEKREG-GSGLSAYPDDPEAAA
NCU03713.1	VQYVSMIDAGSTGSRIHVYKFNNCGPAPELEGEVLFKMTAKIEGQSSGLSAYKDDPLAAA
FG05143.1 	VQYVLMIDAGSTGSRIHVYKFNNCGPTPELEHEE-FKMTEKEVG---GLSNYKGNPVAAA
AN1082.1  	VQYAMMIDAGSQGSRIHVYRFNNCGPTPELEHEE-FKQTEPKEG-GSGLSSYKEDAEGAA
          	***. ****** *******:*****.:**** *  ** *    * .:*** *  :. .**

MG08146.1 	KSLDVLMEAAVKEVPEKLRSCTPVAVKATAGLRLLGDVKSGKILEAVRRHLENDYPFPVV
NCU03713.1	KSLDPLMQAALEVIPDKLKACSPVAVKATAGLRLIGPEKSQKILDAVREHLENDYPFPVV
FG05143.1 	KSLDPLLKVAMDTVPNALKGCTPVAVKATAGLRMIGKEAADAILDEVRRHLEQDYPFPVV
AN1082.1  	KSLDPLMAVAMSTVPDEYKSCSPIAVKATAGLRMLGSELSEKILEAVRTRLETVYPFPVV
          	**** *: .*:. :*:  :.*:*:*********::*   :  **: ** :**  ******

MG08146.1 	ADQ-SGVSIMDGKDEAIYAWVTVNYLLGKIGGPDKSETAAVLDLGGGSTQIVFEPTFKGA
NCU03713.1	SKEEHGVAMMDGADEGVYAWITVNYLLGKIGGPDHSPTAAVFDLGGGSTQIVFEPTFEGL
FG05143.1 	DKENEGVVIMDGSLEGVYAWITTNYLLGKIGGPDKSETAATFDLGGGSTQIVFEPTFKGA
AN1082.1  	SREKGGVEIMDGKDEGVYAWITTNYLLGKIGGPDETPTAAVFDLGGGSTQIVFQPTFEKS
          	  :. ** :***  *.:***:*.***********.: ***.:***********:***:  

MG08146.1 	PNGGMPTKLAEGDHKYELQFGGRHFELYQHSHLGYGLMSAREAIFKALIDEIYSSKK-GD
NCU03713.1	ASGGMPQKLADGDHKYELNFGGRKFDLYQHSHLGYGLMSARDAIFATLVNDLFELNK-QD
FG05143.1 	ADGGMPEKLAEGDHKYDLDFGGRHFELYQHSHLGYGLMAARKAIHGALIKDLTASKG-AD
AN1082.1  	PSGGMPEHLAEGDHKYDLQFGGRHFELYQHSHLGYGLMAARDAVHKAIVEAKLASMPPDD
          	..**** :**:*****:*:****:*:************:**.*:. :::.       . *

MG08146.1 	KSWLNAPVVNPCFGAGMTKTIKVKFGE---GHPLGKEMEINMTGPKSGAP---AQCRALA
NCU03713.1	KSWMQKPVINPCFSTGMTKTVKVPLGD---SHPLGPKVELNMTGPSTPAP---ALCRSLA
FG05143.1 	KSWLKEPIVNPCIAPGMTKAVNVTLDG---SEDL---TLVTFTGPSQPAP---AQCRNLA
AN1082.1  	RSWLKQPISNPCIGPGMEREVKLKYEGEQANHPLAPGVTVKMVGPKEGAPSTAAQCRGLT
          	:**:: *: ***:..** : :::    ..:.. *.    :.:.**.  **:::* ** *:

MG08146.1 	EKILKKDAECKLAPCSFNGVHQPSIAKTFAKEDMYFLSYFTDRAEALGMPESFSLKEMRD
NCU03713.1	ERILKKDAECKLAPCSFNGVHQPQIAKTFAREDIFLLSYFTDRTQPLGMPESFTLREMHD
FG05143.1 	EKILKKDEKCDLAPCSFNGIHQPLLSKTFSKEDVYIFSYFFDRTKPLGMPDSFTLREMHD
AN1082.1  	EKILNKDAACTLAPCSFNGVHQPSLEKTFAKEDVYIFSYFYERTQPLGMPDSFTLDELHD
          	*:**:**  * ********:*** : ***::**::::*** :*::.****:**:* *::*

MG08146.1 	LAEQVCGGKESWGVFSGVKGALEELEGRPEYCLDLNFMLALVHTGYEMPIDREVKVAKKI
NCU03713.1	LAAQVCGGEQSWDVFTSVPGALEELSDRPEHCMDLNFMLALTHTGYEMPIDREVRIAKKI
FG05143.1 	LTQTVCMGKTAWDVFTTVPEAMTELNDRPEWCLDLNFMMALLHTGYDMPIDREVKIAKKI
AN1082.1  	LTKTVCAGESAWSVFEGIPDALKELRDRPEWCLDLNFQLGLLHTGYEMPLSREVKIAKKI
          	*:  ** *: :*.**  :  *: ** .*** *:**** :.* ****:**:.***::****

MG08146.1 	KGNELGWCLGAS2LPLLAKGSGWTCKIAEVH
NCU03713.1	KDNELGWCLGAS2LPLLSQSSGWKCKIKEIH
FG05143.1 	KGNELGWCLGAS2LPLLAGGSGWSCKVNQIA
AN1082.1  	KGNELGWCLGAS2LPLLSQESGWTCRVKEIS
          	*.********** ****:  ***.*:: ::
```
